# Emergence of Micronuclei and Their Effects on the Fate of Cells under Replication Stress

**DOI:** 10.1371/journal.pone.0010089

**Published:** 2010-04-08

**Authors:** Koh-ichi Utani, Yuka Kohno, Atsushi Okamoto, Noriaki Shimizu

**Affiliations:** Graduate School of Biosphere Science, Hiroshima University, Hiroshima, Japan; Oregon State University, United States of America

## Abstract

The presence of micronuclei in mammalian cells is related to several mutagenetic stresses. In order to understand how micronuclei emerge, behave in cells, and affect cell fate, we performed extensive time-lapse microscopy of HeLa H2B-GFP cells in the presence of hydroxyurea at low concentration. Micronuclei formed after mitosis from lagging chromatids or chromatin bridges between anaphase chromosomes and were stably maintained in the cells for up to one cell cycle. Nuclear buds also formed from chromatin bridges or during interphase. If the micronuclei-bearing cells entered mitosis, they either produced daughter cells without micronuclei or, more frequently, produced cells with additional micronuclei. Low concentrations of hydroxyurea efficiently induced multipolar mitosis, which generated lagging chromatids or chromatin bridges, and also generated multinuclear cells that were tightly linked to apoptosis. We found that the presence of micronuclei is related to apoptosis but not to multipolar mitosis. Furthermore, the structural heterogeneity among micronuclei, with respect to chromatin condensation or the presence of lamin B, derived from the mechanism of micronuclei formation. Our study reinforces the notion that micronucleation has important implications in the genomic plasticity of tumor cells.

## Introduction

The integrity or completeness of genomic information is one of the fundamental pre-requisite for life. To maintain this integrity, cells have many elegant mechanisms, many of which have been the subject of recent studies. In human tumor cells, some part of one, or more, of these mechanisms is disrupted, thus genomic integrity is easily broken down. This critically contributes to the malignant transformation of cells. One of the manifestations of such genomic instability is the amplification of oncogenes or drug-resistant genes. Our studies, along with those of others, have previously reported that the amplified genes on extrachromosomal double minutes (DMs) might be eliminated from the cell via inclusion into cytoplasmic micronuclei [1], [2], [3]. This process is accelerated by treatment with hydroxyurea (HU) at a concentration low enough not to completely inhibit DNA replication [4]. Under such replication stress, DNA damage is induced throughout the genome. This causes aggregation of DMs, defects in their mitotic segregation and their emergence as the cytoplasmic micronuclei at the following interphase [5], [6]. The DMs in the micronuclei are active in transcription when surrounded by the nuclear lamina [7] and can still contribute to the malignant phenotype of cells. The content of micronuclei is generally believed to be removed from the cells, but the mechanism for this is not clear.

It is well established that micronuclei are formed from the entire chromosome or from a fragment of it [8], [9], [10], [11]. Such micronuclei are induced by genotoxic stress such as clastogen or aneugen. Micronuclei induction by clastogen involves the induction of either chromosome fragments that lag behind the separating chromosomes or a chromatin bridge between chromosomes at the anaphase of mitosis. On the other hand, aneugen induces the whole chromosomes that were not bound to the mitotic spindle at anaphase, probably by disrupting the spindle checkpoint. Such chromatin is separated from the newly forming nucleus and forms an independent nucleus-like structure, the micronucleus. Therefore, methods to measure the frequency of micronuclei are widely used in genotoxic tests that are used to measure efficacy of newly developed pharmaceuticals or used to diagnose malignant disease [12], [13]. Apart from micronuclei, genotoxic stress also induces several other nuclear abnormalities including nuclear buds that are also called “nuclear protrusions” or “blebs” [11], [14], [15], [16]. Some studies suggest that nuclear buds might be converted into micronuclei during interphase [4]. Taken together, micronuclei and nuclear buds are important indicators for genome instability. Furthermore, they represent interesting biological phenomena in themselves because they may provide clues to understand mechanisms of nucleus reconstruction after mitosis.

The mechanism of micronucleus formation has been repeatedly examined using fixed cells, however, this does not provide the full picture of this dynamic process. Some recent studies have performed time-lapse analyses of micronucleation with live cells [17], [18]. However, further studies are required to clearly understand the complexities of the micronucleation process. In addition, this type of chronological analysis will also address areas that have not yet been studied, i.e. the fate of micronuclei in cells and the fate of cells bearing micronuclei. One demerit of the living cell time-lapse experiments is that only a limited number of cells can be analyzed during a single, time-consuming, experiment. Therefore, we carried out numerous time-lapse experiments in order to analyze a large number of cells and determine the frequency of events semi-quantitatively.

## Results

### Emergence of nuclear abnormalities under replication stress revealed by time-lapse studies

**Figure 1:**
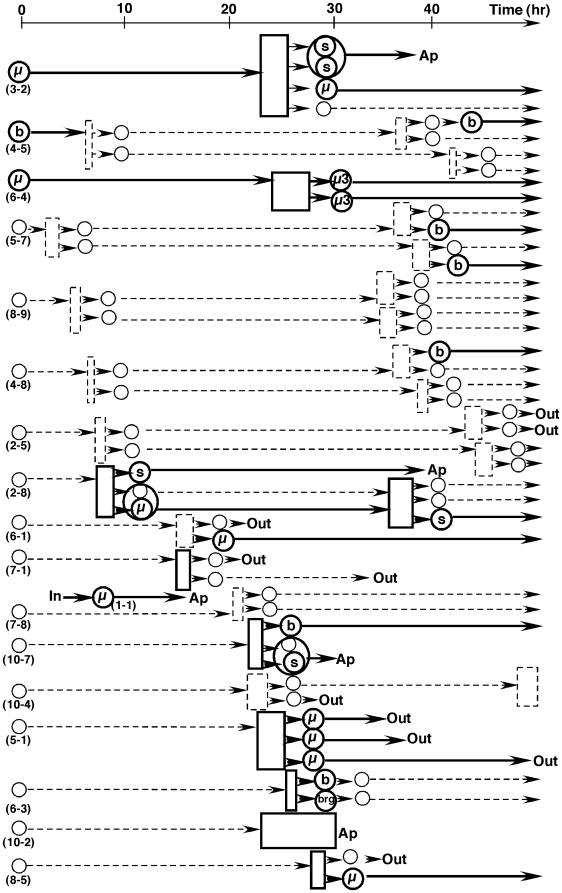
Chronological chart that summarizes a representative time-lapse experiment. Cells were seeded in poly-D-lysine coated glass-bottom dishes and HU was added to the culture (150 µM). After 24 hours time-lapse image acquisition was started for this representative experiment. Six images (1.4 µm apart in the z-axis) were taken for both green fluorescence and phase contrast at 20 min intervals over 48 hours. Each nucleus was numbered (in parenthesis at time 0) by its position on the gridded field at the start time and is indicated by a circle in the figure. Cells with a micronucleus (µ) or buds (b) are noted in appropriate circles. Mitosis is marked as a rectangle, whose width corresponds to its duration. Tripolar or tetrapolar mitoses are drawn as three or four arrows emanating from the mitosis rectangles. As a result of multipolar mitosis, a multinucleated cell (surrounded by a larger oval) or a small nucleus appeared frequently. During the experiment, some cells moved out of the field (out) and some cells entered the field (in). Normal events are shown as dashed lines, whereas abnormal events are highlighted in bold lines. Ap, apoptosis; s, small nucleus; brg, chromatin bridge.

**Figure 2:**
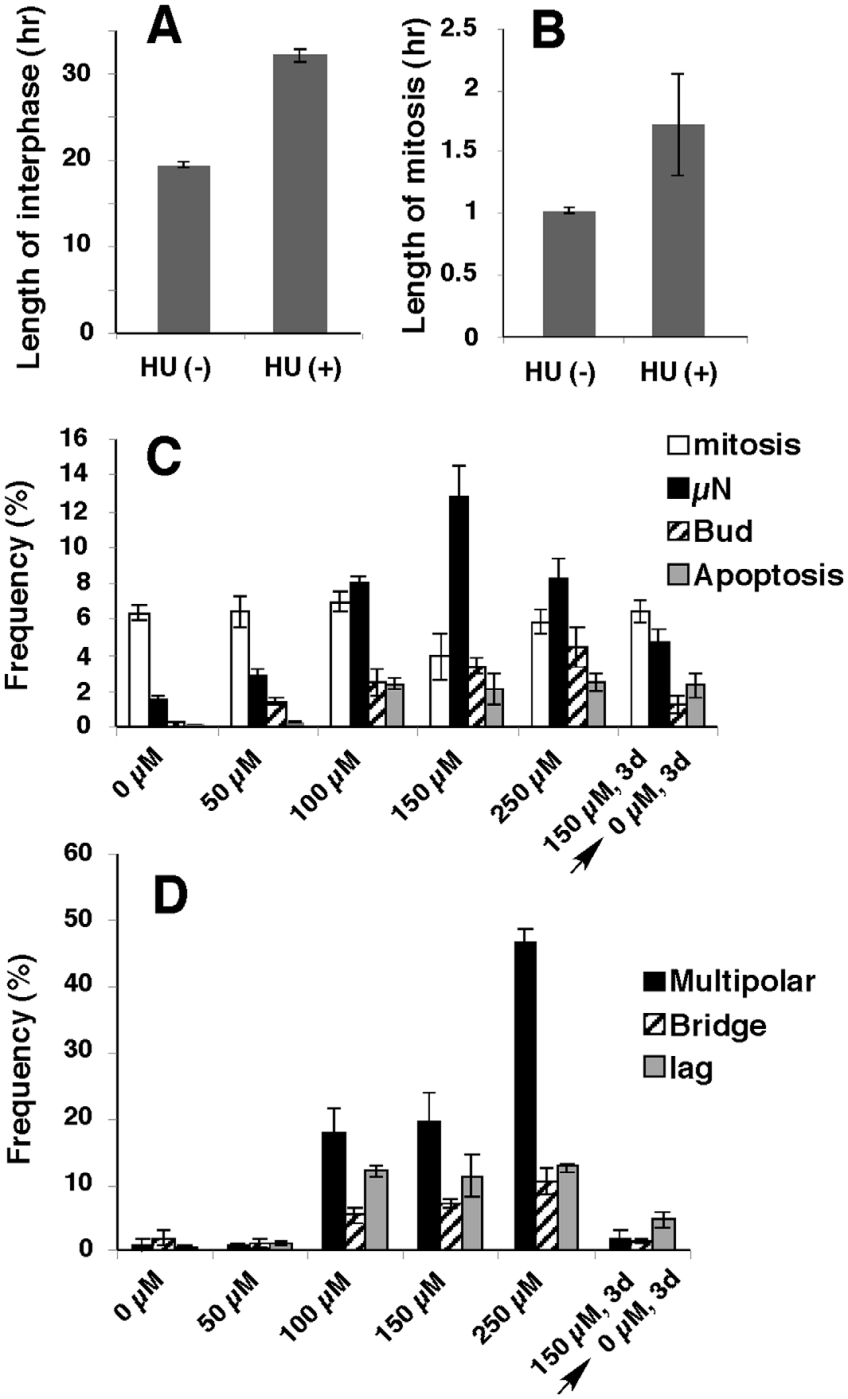
Low concentrations of HU induced delay in cell cycle progression and accumulation of nuclear and mitotic abnormalities. HeLa H2B-GFP cells were cultured in the absence or presence of 150 µM (A, B) or other indicated concentrations of HU (C, D). From representative time-lapse observations (Figs. 1, S1, S2, S3, S4), the length of interphase (from the completion of mitosis to the initiation of the next mitosis; A) or the length of the mitosis (prophase to telophase) was measured. A total of 135 non-treated and 72 HU-treated cells (A), and 158 non-treated and 73 HU-treated cells (B) were examined. Cells were fixed by PFA (C, D) and frequencies of mitotic cells, micronucleated cells, cells bearing nuclear buds and apoptotic cells were obtained by viewing triplicates of 1,000 cells for each sample (C). For D, frequencies of cells showing multipolar mitoses, cells bearing anaphase chromatin bridges or lagging chromatin were obtained by viewing triplicates of >150 mitotic anaphase/telophase cells for each sample. All error bars represent one standard deviation.

In order to study the emergence of nuclear abnormalities including micronucleation after replication stress, we carried out 57 independent 16 to 72 hour time-lapse observations of HeLa H2B-GFP cells in the presence of low concentrations (150 µM) of HU (Fig. 1, S2, S3, S4 and Movies S1 and S2). Many abnormal events were identified and these abnormalities became frequent 24 hours after the addition of HU (Fig. S2), and at subsequent time points (Figs. 2, S3, S4). These events included multipolar mitoses, mitoses with chromatin bridges between separating chromosomes, generation of micronuclei or nuclear buds, and apoptosis, which was identified by chromatin condensation and nuclear fragmentation. In particular, the results suggested that cells bearing micronuclei frequently underwent abnormal mitoses, producing daughter cells with many disorders (for example, see cells 3-2, 6-4 and 2-8 in Fig. 1). Disorders were delivered to descendent cells and typically amplified. In contrast, cells with nuclear buds generally did not result in such catastrophic consequences after mitoses (for example, see cell 4-5 in Fig. 1). Some cells showed no apparent abnormality until 72 hours after the addition of HU (see cells 5-7, 8-9 and 4-8 in Fig. 1). The other finding from a series of these experiments was that the behavior of most micronuclei could be followed for a long time. Some micronuclei could be followed for the duration of one cell cycle, from one mitosis to the next (for example, the daughter cells from cell 2-8 in Fig. 1 and cell 6-5 in Fig. S3). Therefore, micronuclei in HeLa H2B-GFP cells were relatively stable.

As a control, we repeated similar five independent time-lapse observations in the absence of HU. By comparing these two types of experiments, we found that the average length of interphase (Fig. 2A) as well as the length of mitosis (Fig. 2B) were increased by the presence of 150 µM HU, which suggests transient cell cycle arrest by HU-induced DNA damage [6]. Consistent with this notion, the frequencies of interphase cells with nuclear abnormalities and apoptosis (Fig. 2C), as well as the frequencies of abnormal mitosis (Fig. 2D) increased dramatically in an HU-concentration dependent manner. The observed decrease in micronuclei frequency at 250 µM HU (when compared to 150 µM HU) may be explained by growth arrest caused by higher HU concentration. If cells were cultured in the absence of HU for 3 days after they were treated with 150 µM HU for 3 days, some of the nuclear abnormalities still remained but mitotic abnormalities disappeared. These data suggest that most of the nuclear as well as the mitotic abnormalities seen in the HU-treated cultures were induced by replication stress caused by low HU concentrations.

### Formation of micronuclei after replication stress

**Figure 3:**
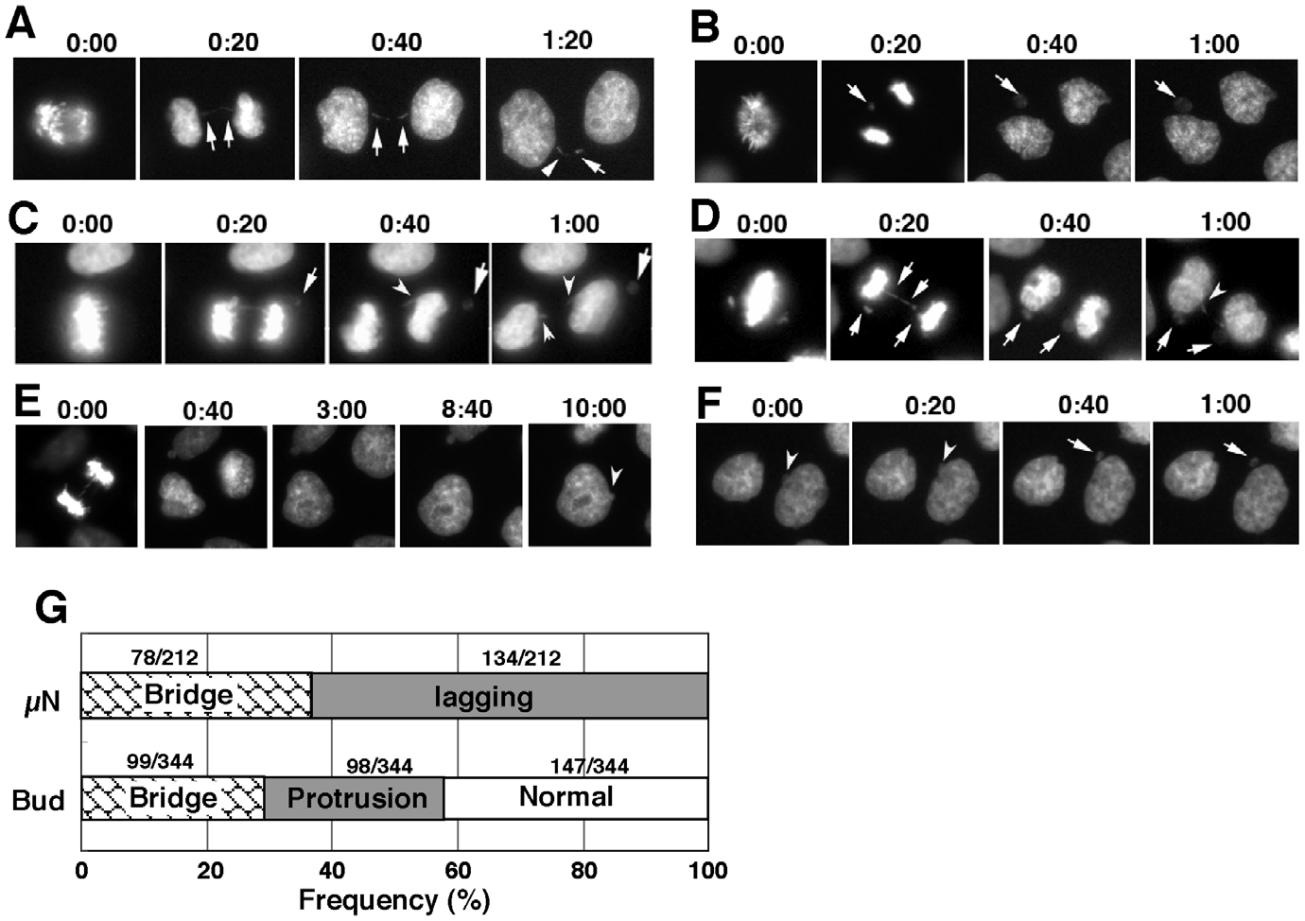
Formation of micronuclei or nuclear buds after replication stress. Micronuclei (indicated by arrows) were formed after formation of a chromosomal bridge between separating anaphase chromosomes (A), from a lagging chromatid that was located between anaphase chromosomes (B) or a chromatid located closer to the spindle pole (C). Both chromatin bridges and lagging chromatids can occur in a single cell (D). The nuclear bud (indicated by arrowheads) might form just after mitosis (C and D) or long after mitosis (E; 10 hours in this case). Buds can detach from the nucleus to generate micronuclei (F). Elapsed time (in hours:minutes) is shown above the images. The frequency of bridge- or lagging chromatid-generated buds or micronuclei after completion of mitosis was scored from more than 30 time-lapse movies and plotted in (G). The number of events observed within the first 72 hours after HU addition was noted.

Most micronuclei appeared directly after completion of mitosis, although some appeared long after mitosis was completed (see below). Early micronuclei were derived from either the chromatin bridge between separating anaphase chromosomes (Fig. 3A) or the chromatid detached from the bulk of the chromosomes during the transition from metaphase to anaphase (Fig. 3B). The detached chromatid was located between the separating anaphase chromosomes and may be merotelically attached to the spindle [19]. Unexpectedly, detached chromatids were also located at position closer to the spindle pole than the chromosomes (Fig. 3C) or at the side of the chromosomes (Fig. 3D), possibly reflecting syntelical or monotelical attachment to the spindle. Chromatids located in these positions produced micronuclei. This chromatid is not the “lagging chromatid” in a strict sense, however, we will use this term for all detached chromatids as this term is commonly used for micronuclei formation. The chromatin bridge and the lagging chromatid might simultaneously appear in the same mitotic cell and they can independently generate micronuclei (Fig. 3D). By examining many time-lapse movies captured during the initial 72 hours in the presence of HU, we observed on 212 occasions micronuclei generation just after mitosis. As shown in Fig. 3G, 37% (78/212) and 63% (134/212) of the micronuclei were generated from the bridge and the lagging chromatid, respectively, under these conditions.

A part of the chromatin bridge might remain as the nuclear bud after its resolution (see arrowheads in Fig. 3C and D). Such a mechanism would explain 29% (99/344) of the buds that were detected just after mitosis (Fig. 3G). On the other hand, the buds might also appear at the end of mitosis in the absence of detectable chromatin bridges. This type of bud constituted 28% (98/344) of the total buds. Furthermore, there were buds that were generated long after the apparently normal mitosis, typically by the protrusion of the interphase nucleus (Fig. 3E). This type of bud constituted 43% (147/344) of all buds (Fig. 3G). There were also nuclear buds that appeared to be converted to micronuclei (Fig. 3F). The frequency of such events was quite low under these conditions and is not depicted in Fig. 3G.

### HU increased multipolar mitoses, which frequently generated micronuclei

**Figure 4:**
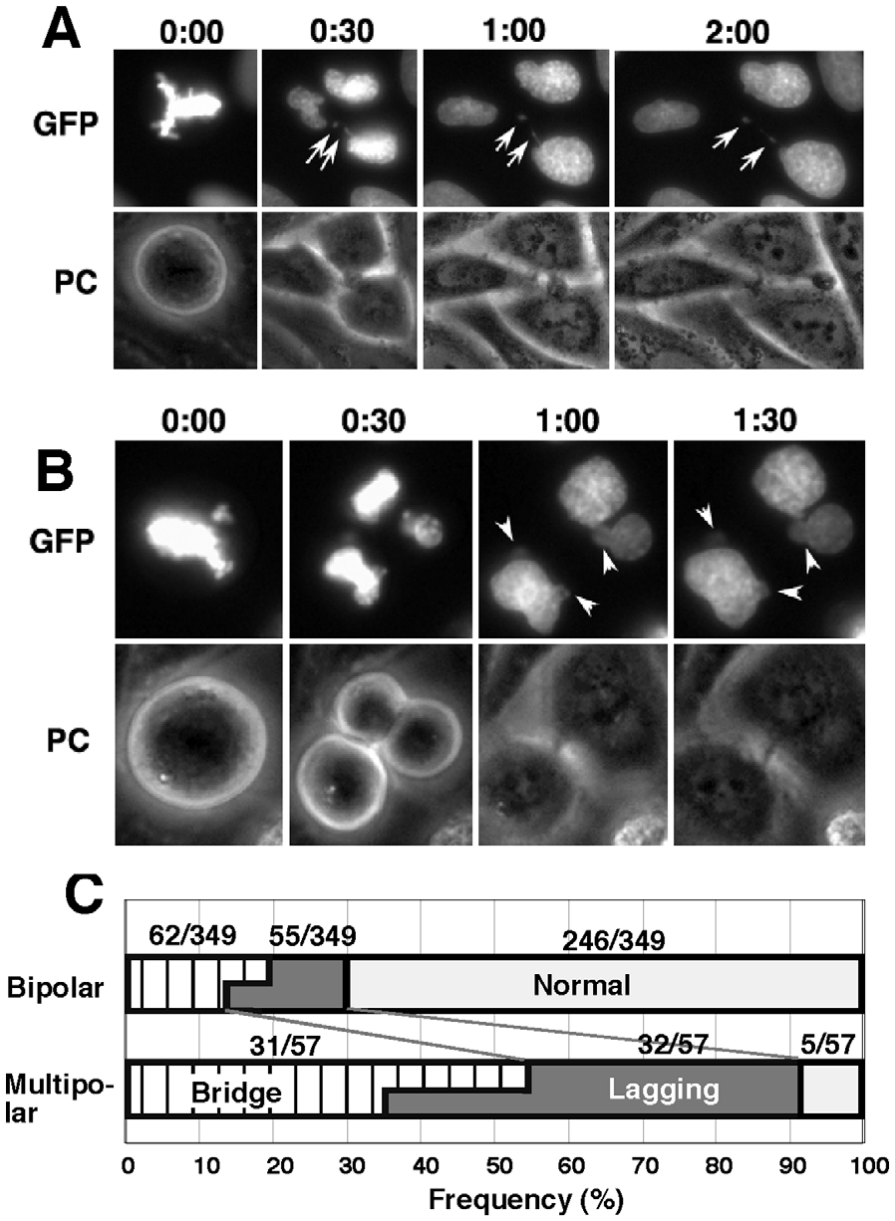
Multipolar mitosis is a frequent cause of micronuclei generation. Representative time-lapse images for a tripolar mitosis that generated a chromatin bridge (A) or a lagging chromatid (B). By examining 349 normal and 57 multipolar mitoses, the frequency of chromatin bridge and/or lagging chromatid occurrence was scored and is summarized in (C). GFP, fluorescent channel; PC, phase contrast.

Under HU-induced replication stress, multipolar mitosis occurred frequently (Fig. 1). It was suggested that HU might result in the over-replication of the centriole by inducing DNA damage and uncoupling the centriole cycle and the cell cycle [20], [21]. Such multipolar mitosis frequently generated the chromatin bridge and/or the lagging chromatid, which resulted in micronuclei formation. The representative time-lapse images for the generation of bridge or lagging chromatid from tripolar mitosis is shown in Fig. 2A and B, and their frequency among the 349 dipolar or 57 multipolar mitoses are summarized in Fig. 4C. Both chromatin bridges and lagging chromatids were generated far more frequently during the multipolar mitoses than the normal bipolar mitoses (Fig. 4C). Formation of micronuclei from multipolar mitoses had been suggested by studies on fixed oral cancer cells [22] and live Chinese hamster cells under phase contrast microscopy [23].

### Multipolar mitoses generated cells with small nuclei that were prone to apoptotic cell death

**Figure 5:**
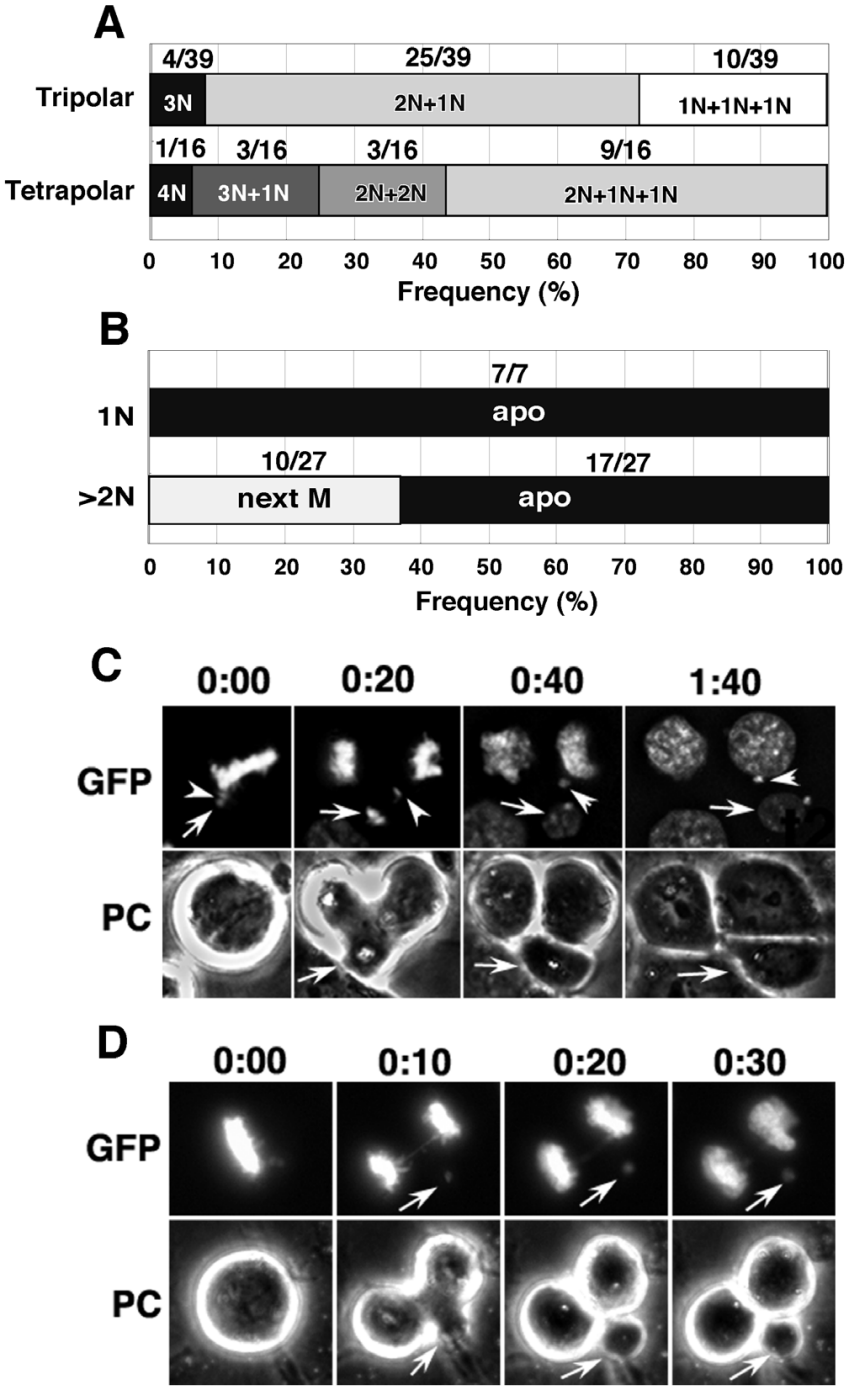
Asymmetrical multipolar division generates hypoploid cells by partial genome elimination. We followed cells produced from 39 tripolar or 16 tetrapolar mitoses and obtained the frequency of mononuclear and multinuclear cells (A). The mononuclear cells produced from the multipolar mitoses underwent apoptosis, whereas a portion (10/27) of the multinuclear cells reached the next mitoses (B). The distribution of genetic material among the multipolar mitoses can be asymmetric. Thus, in an extreme case, one pole might receive only a small amount of chromatin that resembled the lagging chromatid (C and D).

After the completion of multipolar mitoses, cytokinesis did not always separate each daughter nucleus, thus multinucleated cells were frequently generated (Fig. 5A). We found 74% (29/39) of the tripolar and all (16/16) of the tetrapolar mitoses generated multinucleated cells. By following the fate of a portion of these cells, we found that the mononuclear cells from the multipolar mitoses underwent apoptosis during subsequent interphase (7/7), whereas a portion of the multinuclear cells (10/27) could reach the next mitoses (Fig. 5B). The apoptosis of mononucleated cells should not be a result of loss of a specific gene because all cells had undergone apoptosis despite these cells received about two-thirds of the genome. Rather, the total amount of genome complements, i.e. less than diploid, is expected to induce the apoptotic response. We hereafter call such hypoploid daughter nuclei “small nuclei”, in order to discriminate them from micronuclei that originate from acentric chromatin.

On the other hand, the distribution of genetic material during multipolar mitoses can be asymmetric (see Fig. 4A and B). In the extreme, one pole received very little chromatin (e.g. Fig. 5C and D). Interestingly, these images appear similar or identical to lagging chromatids observed at anaphase (Fig. 3B), and they cannot be discriminated by conventional static observation techniques and fixed cells. However, we observed that the small nucleus was separated by cytokinesis, which generates a small cell that either fused with one of the sister cells or underwent apoptosis, as seen for multipolar mitosis in general. Thus, apoptosis of the small cell might eliminate the apparently lagging chromatid.

### The fate of micronucleated cells

**Figure 6:**
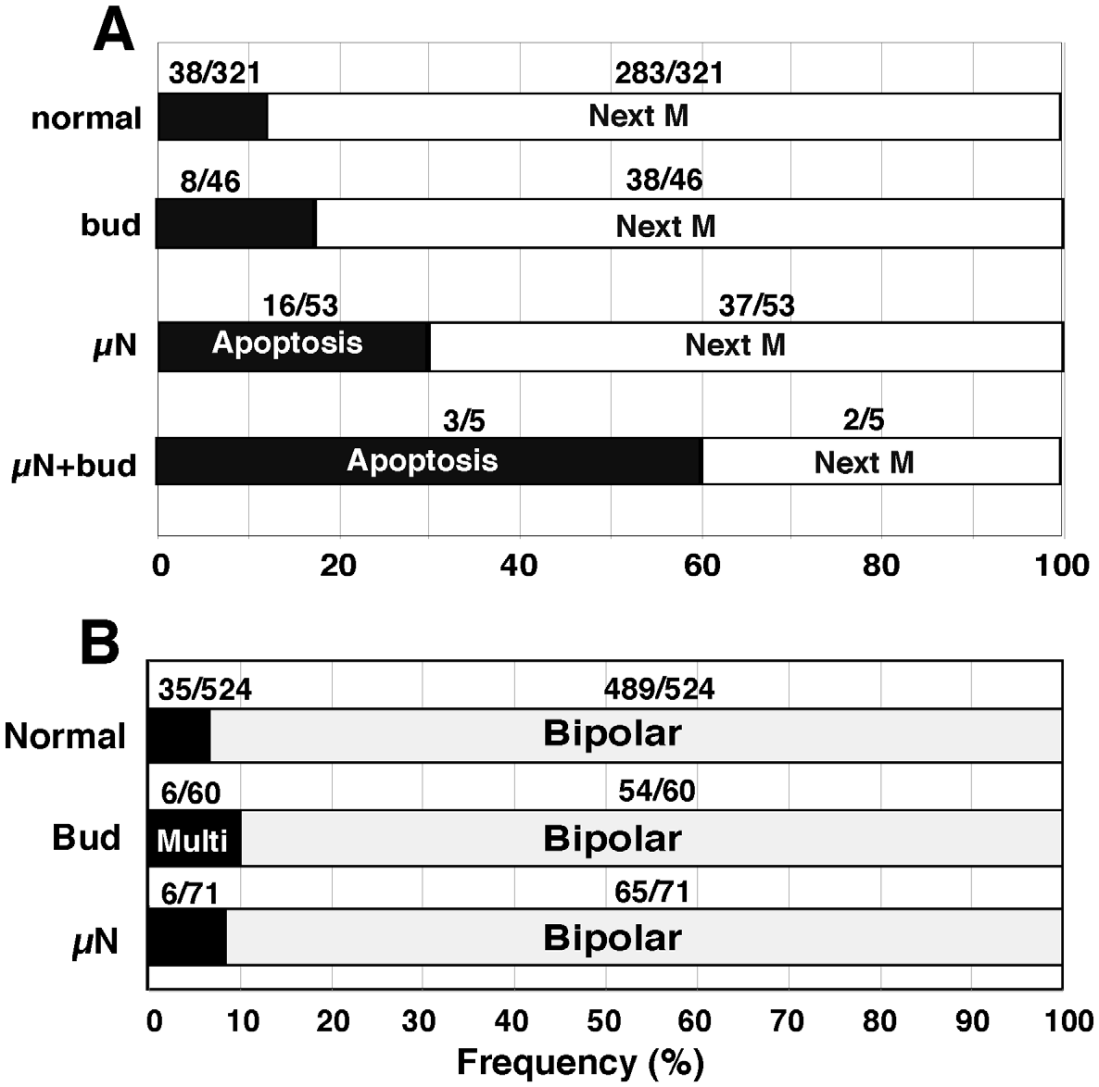
Micronuclei-bearing cells frequently underwent apoptosis but not multipolar mitosis. By examining more than 20 time-lapse movies, each of which captured more than 20 cells for over 48 hours, we followed the interphase cells bearing micronuclei and/or nuclear buds, and counted the frequency of cells that underwent apoptosis until they entered the next M phase (A) or the frequency of cells that underwent multipolar mitoses (B). The cells frequently underwent apoptosis if they contained micronuclei, buds, or especially both (A). However, the frequency of multipolar mitoses was similar between the cells with or without micronuclei or buds (B).

During the course of time-lapse observation, we found that the frequency of apoptotic cell death was much higher among the cells bearing micronuclei (16/53 = 30%) compared with cells bearing normal nuclei (38/321 = 12%, see Fig. 6A). This frequency was highest if cells had both micronuclei and buds (3/5 = 60%). On the other hand, if the cells bearing micronuclei or buds reached mitoses, the frequency of multipolar mitoses (6/71 = 8.5%) was almost the same as for cells with normal nuclei (35/524 = 6.7%, Fig. 6B). Therefore, presence of micronuclei was frequently associated with apoptosis but did not result in multipolar mitosis.

**Figure 7:**
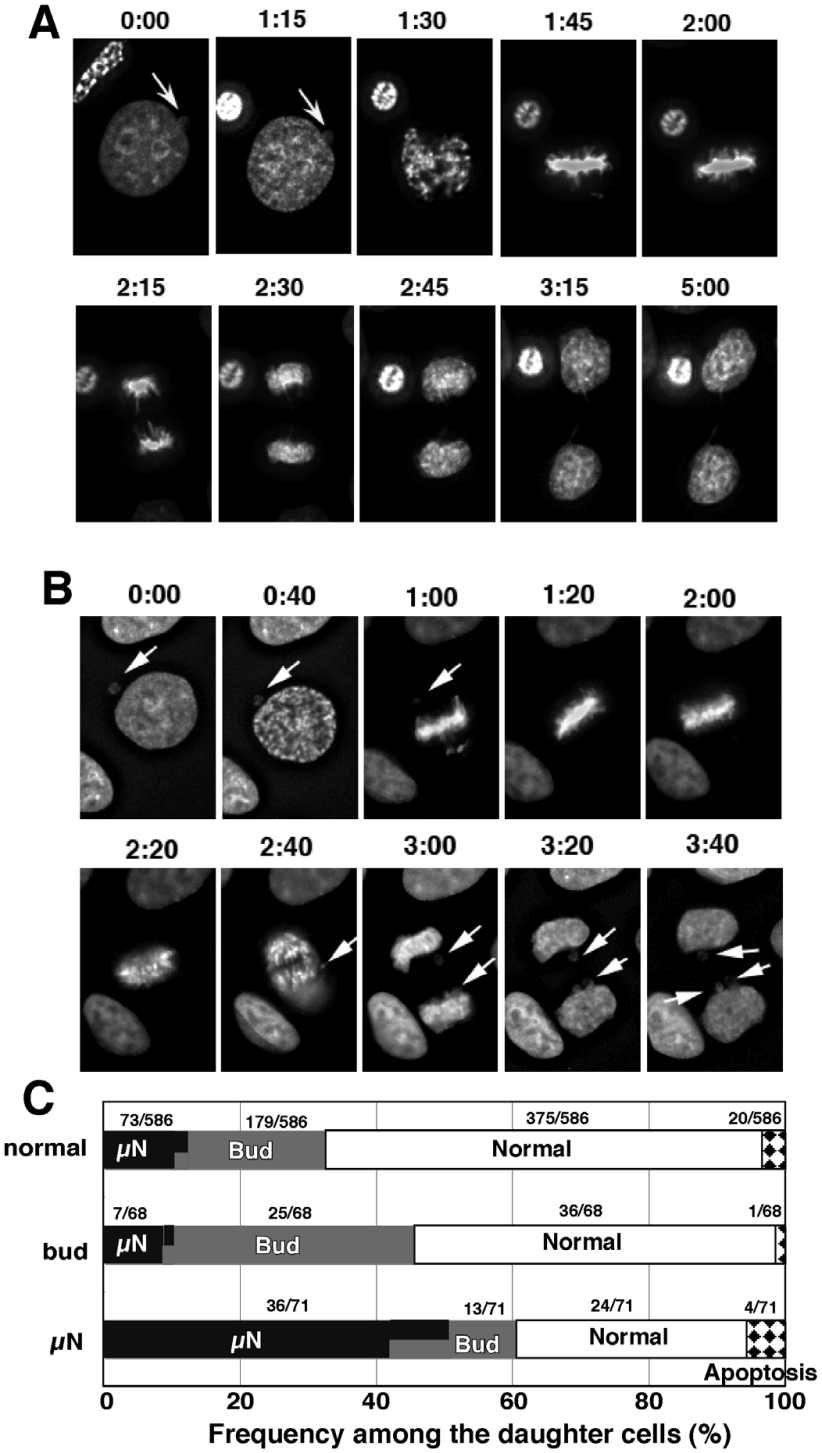
The micronucleus was diminished or amplified after the cells passed through mitosis. Two representative time-lapse images for the mitosis of micronucleus-bearing cells are shown in (A) and (B). In (A) the daughter cells did not bear a micronucleus (MN), whereas in (B) the daughter cells had many micronuclei. Among more than 30 time-lapse movies, we followed the cells with or without buds or micronuclei during mitoses, and recorded the frequencies of daughter cells with micronuclei or buds (C). The horizontal split of the bars between MN and buds indicates the fraction of daughter cells bearing both micronuclei and bud. In the graph, numbers of each event among the daughter cells were noted.

If cells bearing micronuclei entered mitoses, most of the micronucleus was difficult to trace among condensing chromatids of nuclear origin (Fig. 7A and B). After anaphase, there were cells in which the micronucleus did not appear in both daughter cells (Fig. 7A) until more than 10 hours after completion of mitosis (cell 4-2 in Fig. S4). We were unable to determine whether the micronucleus was eliminated from the cells during mitosis or whether it re-entered the main nucleus. There were many cases in which larger numbers of micronuclei than those in the previous G2 phase appeared in daughter cells (see Fig. 7B; frequencies summarized in Fig. 7C). This showed that mitosis of micronuclei-bearing cells resulted in the production of micronucleated cells at much higher frequency than those in cells with a normal nucleus or those containing buds (Fig. 7B).

### Structural heterogeneity among micronuclei depends on their biogenesis

**Figure 8:**
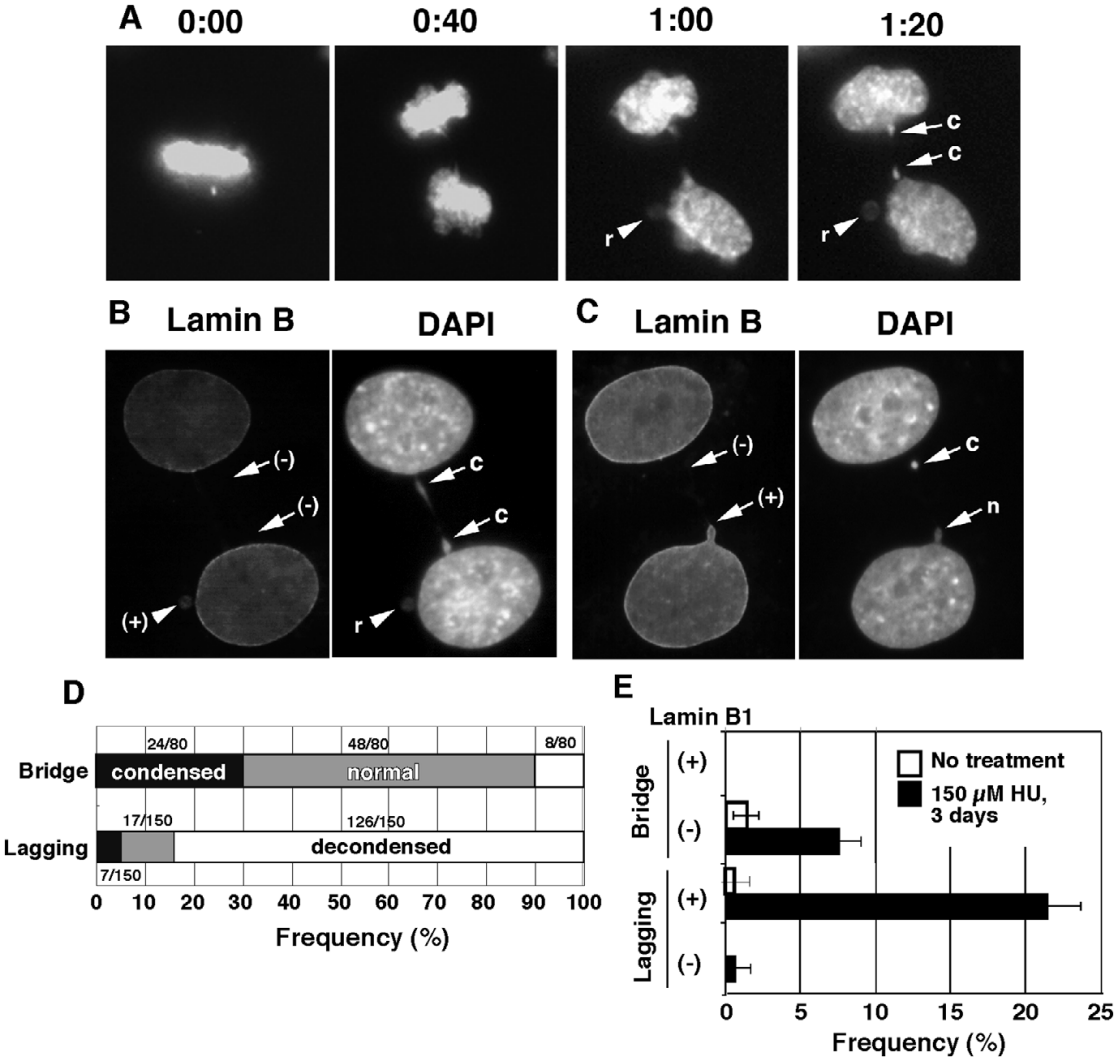
The anaphase bridge-derived micronuclei had condensed chromatin and were devoid of nuclear lamin B. (A) A series of time-lapse images for the H2B-GFP fluorescence shows that anaphase bridge-derived micronuclei (arrows) had condensed chromatin (c), whereas lagging chromatid-derived micronuclei had more relaxed chromatin (r; arrow head) and buds had normal condensation level (n) as the nucleus. (B, C) Among fixed wild-type HeLa cells, lamin B was detected by immunofluorescnce and DNA was counterstained with DAPI. The lagging chromatid-derived micronuclei seen in (B) (arrowhead) had both relaxed chromatin and lamin B (+), whereas the bridge-derived micronuclei had condensed chromatin that lacked lamin B (−) (see arrows in B and upper arrow in C). The lower arrow in (C) indicates a bud that was positive for lamin B stain. The frequencies of each of these cases were recorded and plotted in D and E. Data in (E) were obtained by scoring quadruplicates of more than 100 telophase cells for each sample.

It was known that micronuclei were structurally heterogeneous with respect to the chromatin condensation level and the presence of nuclear lamina [5], [7], [24], [25], however, the reason for this had not been determined. In this study we found that one factor contributing to heterogeneity was the mechanism of generation of micronuclei. The chromatin condensation level was quite different between micronuclei generated by different mechanisms. In our study, chromatin condensation was roughly estimated by the intensity of fluorescence from H2B-GFP or DAPI stain, because these intensities reasonably correlate with chromatin condensation, e.g. heavier label or stain at heterochromatic regions. Representative time-lapse images illustrate that chromatin in the lagging chromatid-derived micronuclei was relaxed compared to that in the main nucleus during telophase (Fig. 8A; at 1:00) to early G1 phase (at 1:40, indicated by the arrowhead). On the other hand, chromatin bridges also generated micronuclei in the same cells (indicated by the arrows), and here chromatin was more condensed compared to the main nucleus even after the completion of mitosis (Fig. 8A). In order to obtain frequencies (Fig. 8D), we examined many cells that were fixed by PFA *in situ*. We identified chromatin-bridge-derived micronuclei based on the criterion that the bridge usually produced multiple micronuclei or buds, which appear “in line” inside the anaphase/telophase cells (see Figs. 3A, D and 8A). The micronuclei that did not pass this criterion were classified as lagging chromatid-derived. Our results suggest that the micronuclei generated from the lagging chromatid generally had more relaxed chromatin than the nucleus, whereas those generated from the bridge had more condensed chromatin.

Presence of lamina around micronuclei had previously been correlated to transcription inside micronuclei [7], which has important implications for cell phenotype. Thus, we imaged presence of lamin B among fixed cells by immunofluorescence. We distinguished chromatin bridge-derived and lagging chromatid-derived micronuclei according to the criterion stated above. We found lamin B around lagging chromatid-derived micronucleus (Fig. 8B, arrowhead) but rarely around bridge-derived micronuclei (Fig. 8B, two arrows). Another representative image (Fig. 8C) shows a bridge-derived chromatid as condensed and devoid of lamin B while apart from the nucleus (upper arrow), but surrounded by lamin B when connected to the nucleus as a nuclear bud (lower arrow). We recorded the frequency of each type of micronuclei in the fixed and lamin B immunostained cells (Fig. 8E). The results suggested that the bridge-derived micronuclei were less frequently associated with lamin B protein compared to the lagging chromatid-derived micronuclei.

## Discussion

In this study, we have addressed how micronuclei emerge and affect cell fate after replication stress in human cervical cancer (HeLa) cells. We had reported that the same replication stress used here can induce micronuclei in normal cells if p53 is malfunctioning [4]. Therefore, data presented here may also be applicable to p53 negative cells. Here, we found that presence of micronuclei typically resulted in apoptotic cell death but not in multipolar mitosis. There are two possible explanations for these observations: (1) Micronuclei-bearing cells had severe DNA damage that might induce apoptosis. However, cells bearing micronuclei caused multipolar mitosis as rarely as normal cells. As centriole over-replication is a likely outcome of DNA damage, it seems plausible that micronucleated cells had overcome HU-induced damage before they reached mitosis. (2) Our favored explanation is that the malfunctioning of gene expression in the micronuclei might trigger apoptosis. We suggested previously that genes in lamin B-negative micronuclei were not expressed, whereas genes in lamin B-positive micronuclei were actively expressed and might cause irregularities that affect cell phenotypes [7]. Thus, entrapment of genes in the micronuclei may have severe phenotypic effects on the cells, which may trigger apoptosis.

It has generally been thought that the contents of micronuclei disappear from cells, although the mechanism for this has not been determined. Revealing this mechanism has important implications because it provides a mechanism for loss of genetic material from cells. Furthermore, the elimination of DMs carrying amplified oncogenes was associated with their micronuclear entrapment [1], [2], [3]. For human colorectal carcinoma COLO 320DM cells, micronuclei enriched with DMs and surrounded by lamin B were found in the culture medium [26]. Time-lapse observation of cells bearing GFP-tagged DMs suggest that micronuclei enriched with DMs were extruded from the cells by cytoplasmic membrane blebbing (K. Utani *et al.*, manuscript in preparation). In the current study with HeLa cells, disappearance of micronuclei during interphase was not apparent. However, some micronuclei seemed to disappear during mitosis because there were micronuclei-bearing cells that generated two apparently normal daughter cells after mitosis (Fig. 7A). Cytoplasmic membrane blebbing was very active during mitosis of these cells (data not shown), and may thus be involved in elimination of micronuclei. On the other hand, unequal tripolar mitosis generated small chromatids at anaphase, which was very close to lagging chromatids that form in the micronucleus. This phenomenon, despite the low frequency, may provide a mechanism of elimination of the lagging chromatid and the micronucleus, because the small cells with tiny nuclei underwent apoptosis. In addition to this, recent experiments suggest that the content of lamin B-negative micronuclei is not replicated during the entire cell cycle, suggesting the dilution of micronuclear content during cell proliferation (A. Okamoto *et al.*, unpublished observation). Taken together, it appears that the content of micronuclei may be eliminated from the cells by several different, separable mechanisms.

The genetic material was neither transcribed nor replicated in the lamin-less micronuclei [7]. Therefore, the question of why and how the structural heterogeneity of micronuclei arose has important implications. We show here that part of the reason derives from the mechanism of micronuclei generation, as most of the chromatin bridge-derived micronuclei had condensed chromatin that was not associated with lamin B, whereas the lagging chromatid-derived micronuclei had more relaxed chromatin with lamin B. The question of why and how these differences arise between micronuclei is important because the answer will contribute towards an understanding of how the nucleus is re-constructed after the completion of mitosis. Our future studies will address this question.

## Methods

### Cell culture

HeLa H2B-GFP cells were obtained from Dr. Teru Kanda (Hokkaido University). These cells express a fusion protein of histone H2B and GFP; as a result the entire chromatin in all cells emits bright green fluorescence [27]. The cell cycle progression of these cells does not differ from the parental HeLa cells in all aspects examined [27]. In some experiments, we used HeLa cells without the transgene that were obtained from the Cell Resource Center for Biomedical Research, Tohoku University. For both HeLa cell lines, cells were grown and maintained in DMEM medium (Nissui Pharmaceutical Co., Tokyo) supplemented with 10% fetal calf serum and incubated at 37°C in 5% CO_2_ with constant humidity.

### Time-lapse microscopy of live cells

Cells were cultured in poly-D-lysine coated glass bottom dishes (35 mm diameter, MatTek Co., Ashland, MA). Hydroxyurea (HU) (Sigma, St. Louis, MO) was added to the culture at 150 µM. This concentration of HU induced many gamma H2AX foci within 1 hour in the S phase nucleus of human colorectal carcinoma COLO 320DM cells [6]. The cells in the dish were cultured on the microscope stage by using a stage top incubator system (ONIVF; Tokai Hit Co., Japan) with a control unit (INU; Tokai Hit Co.) in a constant flow of air containing 5% CO_2_ at 37°C with constant humidity. The incubator system was equipped with a Nikon inverted microscope (TE2000-E, Nikon, Tokyo), which incorporates a high precision motorized-focus and vibration-free optical path changeover mechanism that facilitates image capture in 3D. Both the microscope and the CCD camera (DS, Nikon) were controlled by NIS-element software (Nikon). The epifluorescence images were captured using the Precentered Fiber Illuminator (Intensilight, C-HGFI, Nikon), the ND-filter (64-fold in total), the adequate fluorescence filter set, and 40× objective lens (Nikon Plan Fluor, NA 0.60). In most experiments, six to eight images at 1.4 µm z-intervals were captured at 10 to 20 min time intervals. The acquired images were viewed using the NIS-element software. Some images were deconvolved using the same software. The images were exported from the NIS-element as a TIFF file, and processed using Adobe Photoshop CS version 8.0.1 (Adobe Systems Inc.).

### Immunofluorescent detection of lamin protein in fixed cells

The cells were grown on cover slides and fixed with 3% paraformaldehyde for 10 min at room temperature. Cells were then permeabilized with 0.5% NP-40 in PBS for 10 min at room temperature, rinsed in PBS, and immersed in 100% methanol for 3 min at −20°C. Indirect immunofluorescence was performed using affinity-purified goat polyclonal anti-Lamin B (M-20; Santa Cruz Biotechnology, Inc, CA) that detects lamin B1 and, to a lesser extent, lamin B2 and B3. Bound antibody was detected using Texas Red-conjugated rabbit anti-goat antibody (EY laboratories, San Mateo, CA). The slide was counterstained with DAPI and examined by epifluorescence microscopy (ECLIPSE TE2000-U, Nikon) using a microscope equipped with a 100x objective lens (Nikon Plan Fluor, NA 1.30 oil) and an appropriate filter set. Digital images were acquired with a Fuji FinePix S1Pro digital camera (Fuji Film Co.) and processed with Adobe Photoshop CS, as above.

## Supporting Information

Figure S1 A chart similar to Figure 1. In this case, the cells were grown in the absence of HU. The time-lapse images were obtained at 30 min intervals during 65 hours.
(0.44 MB TIF)

Figure S2 A chart similar to Figure 1. In this case, the time-lapse images were obtained at 20 min intervals during 69 hours, starting from 4 hours after the addition of 150 µM of HU.
(0.18 MB TIF)

Figure S3 A chart similar to Figure 1. In this case, the time-lapse images were obtained at 20 min intervals during 44 hours, starting from 24 hours after the addition of 150 µM of HU.
(0.13 MB TIF)

Figure S4 A chart similar to Figure 1. In this case, the time-lapse images were obtained at 15 min intervals during 26 hours, starting from 48 hours after the addition of 150 µM of HU.
(0.07 MB TIF)

Movie S1 This movie corresponds to the chart shown in Figure 1.
(7.45 MB MOV)

Movie S2 This movie corresponds to the chart shown in Figure S1.
(8.26 MB MOV)
