# Supplementary figures and images for: Emergence of Micronuclei and Their Effects on the Fate of Cells under Replication Stress
Source: PLoS One. 2010 Apr 8;5(4):e10089. doi: 10.1371/journal.pone.0010089 (PMC2851613; doi:10.1371/journal.pone.0010089)

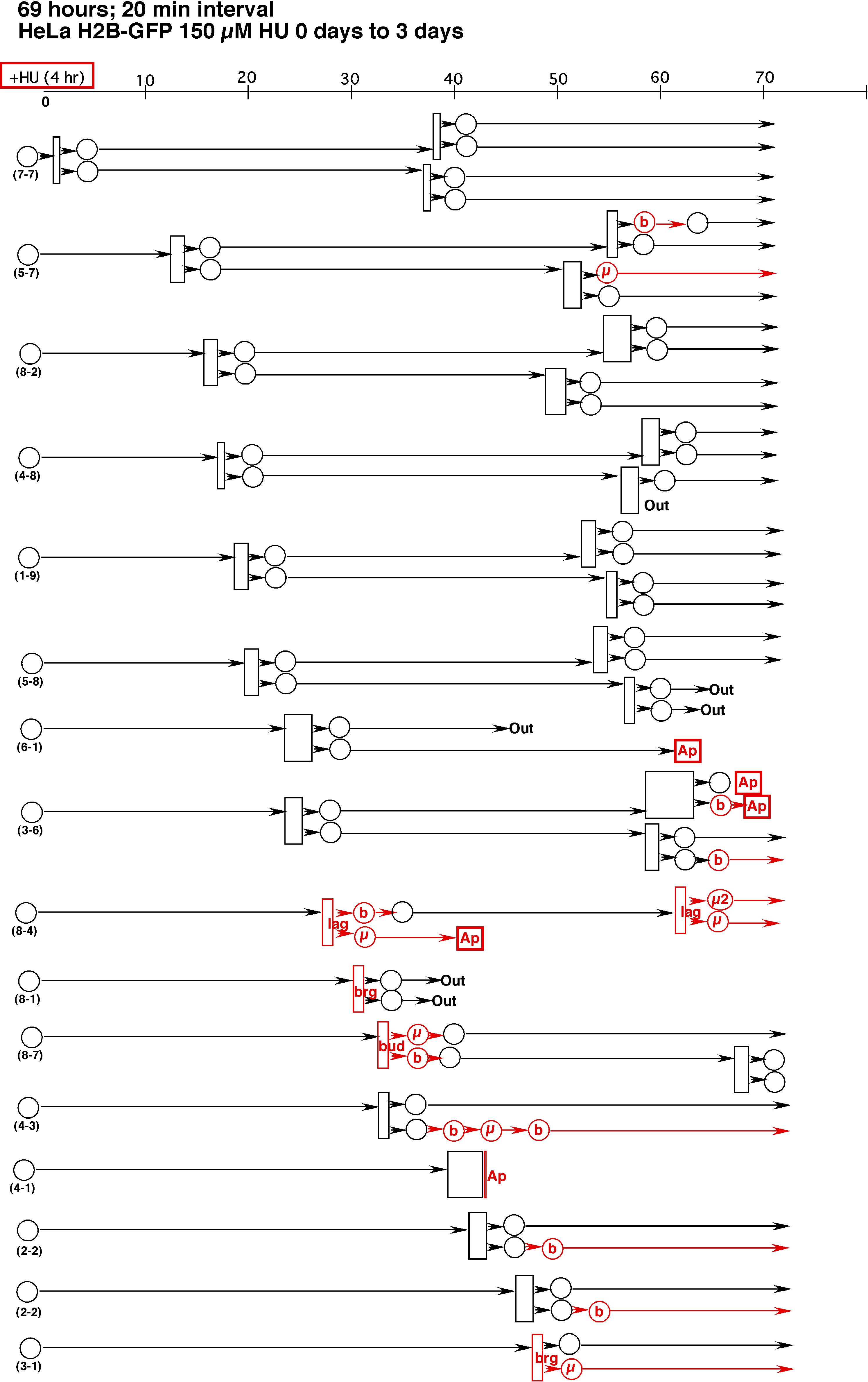

Supplement: Figure S2 — A chart similar to Figure 1. In this case, the time-lapse images were obtained at 20 min intervals during 69 hours, starting from 4 hours after the addition of 150 µM of HU. (0.18 MB TIF) [file pone.0010089.s002.tif]

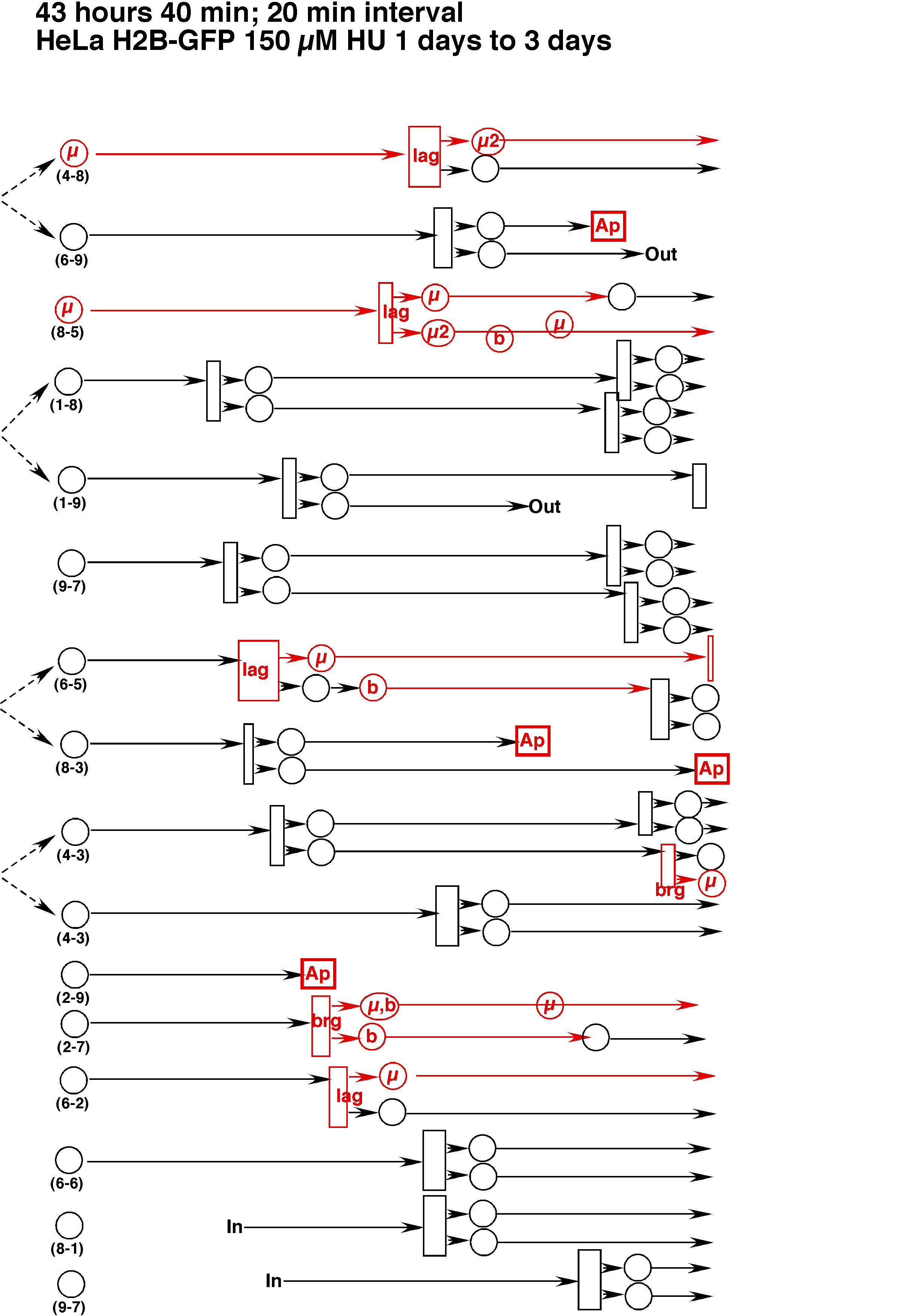

Supplement: Figure S3 — A chart similar to Figure 1. In this case, the time-lapse images were obtained at 20 min intervals during 44 hours, starting from 24 hours after the addition of 150 µM of HU. (0.13 MB TIF) [file pone.0010089.s003.tif]

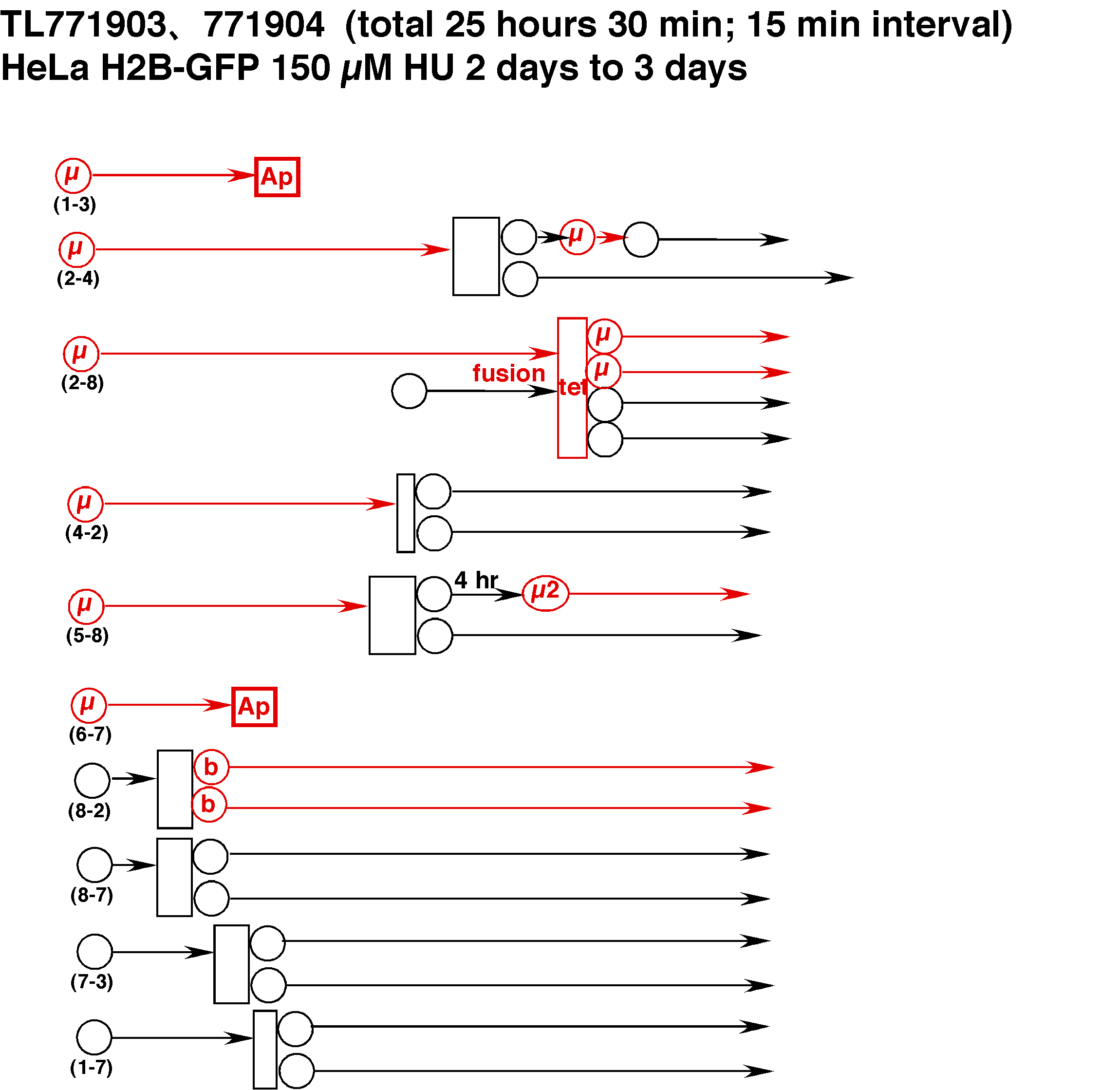

Supplement: Figure S4 — A chart similar to Figure 1. In this case, the time-lapse images were obtained at 15 min intervals during 26 hours, starting from 48 hours after the addition of 150 µM of HU. (0.07 MB TIF) [file pone.0010089.s004.tif]
